# A Tea Polyphenol-Infused Sprayable Thermosensitive Liposomal Hydrogel for Enhanced Anti-Inflammatory and Antibacterial Psoriasis Treatment

**DOI:** 10.3390/jfb16040124

**Published:** 2025-04-01

**Authors:** Wei Shen, Qilian Ye, Hongbo Zhang, Shenghong Xie, Shiqi Xie, Cailian Chen, Jinying Liu, Zhengwei Huang, Hai-Bin Luo, Ling Guo

**Affiliations:** 1 Key Laboratory of Tropical Biological Resources of Ministry of Education, School of Pharmaceutical Sciences, Hainan University, Haikou 570228, China; 22211007000026@hainanu.edu.cn (W.S.); 22211007000029@hainanu.edu.cn (Q.Y.); 23211007000027@hainanu.edu.cn (S.X.); 20223000582@hainanu.edu.cn (S.X.); 22220860020015@hainanu.edu.cn (C.C.); 22211007000008@hainanu.edu.cn (J.L.); 2 Department of Neurosurgery, The Second Affiliated Hospital of Nanchang University, No 1. Mingde Road, Donghu District, Nanchang 330006, China; hongbozhang99@smu.edu.cn; 3 State Key Laboratory of Bioactive Molecules and Draggability Assessment, Guangdong Basic Research Center of Excellence for Natural Bioactive Molecules and Discovery of Innovative Drugs, College of Pharmacy, Jinan University, Guangzhou 510632, China; huangzhengw@jnu.edu.cn

**Keywords:** psoriasis, inflammation, bacterial infection, tea polyphenols, lauric acid, liposomes, sprayable hydrogels

## Abstract

Psoriasis is a chronic and recurrent inflammatory disease driven not only by intrinsic factors such as immune system dysregulation but also by external factors, including bacterial infections. In contrast to the control of a single pathogenic pathway, combination therapies addressing both the immune and infectious components of psoriasis pathogenesis may offer a more effective strategy for controlling its progression. In this study, we developed a sprayable hydrogel incorporating tea polyphenol-loaded lauric acid liposomes (TP@LA-Lipo gel) to investigate its anti-inflammatory and antibacterial role in psoriasis. Our results demonstrated that TP@LA-Lipo modulated macrophage activity, reduced the expression of iNOS and TNF-α, and remodeled the immune microenvironment. Meanwhile, TP@LA-Lipo effectively eliminated *Staphylococcus aureus* and *Escherichia coli* through membrane disruption, mitigating the provoked inflammatory response. More importantly, TP@LA-Lipo gel, when sprayed onto the psoriasis lesions, provided sustained drug release over three days, enabling deeper penetration through the thickened stratum corneum to reach the inflamed layers beneath. Furthermore, in an imiquimod-induced psoriasis mouse model, TP@LA-Lipo gel effectively restored the damaged skin, alleviated histopathological changes, and reduced the systemic immune response. In summary, these findings indicate that TP@LA-Lipo gel offers a comprehensive strategy for effective disease management and improving the quality of life for psoriasis patients.

## 1. Introduction

Psoriasis is an autoimmune disorder characterized by the excessive production of inflammatory cytokines, which initiates a cascade of biological processes. This dysregulated immune response leads to keratinocyte proliferation and epidermal hyperplasia, contributing to the thickening of the skin [1,2]. These changes underlie the development and persistence of the hallmark features of psoriasis, including the formation of red, scaly patches on the skin [3]. In addition to autoinflammatory responses, emerging evidence suggests that infections can serve as environmental triggers for psoriasis, playing a role in its persistence [3,4]. Bacterial infections can provoke an overactive immune response, causing the body to mistakenly identify healthy skin cells as foreign invaders. This misidentification leads to the characteristic inflammation and excessive skin cell proliferation observed in psoriasis [4]. Importantly, bacterial infections can exacerbate the condition by triggering itching and scratching, leading to the disruption of the skin barrier [5]. This disruption facilitates deeper bacterial penetration into the skin, further stimulating the immune system and perpetuating the inflammatory cycle. As a result, the presence of bacteria not only contributes to the direct symptoms of psoriasis but may also complicate the overall condition by initiating or worsening flare-ups [3]. These insights have spurred a growing interest in targeting skin microbiota as a potential therapeutic strategy for psoriasis. Hence, by combining anti-inflammatory agents with antibacterial approaches, there is the potential to develop more effective treatments for psoriasis.

Recent studies have demonstrated the natural properties of tea polyphenols (TP), including their ability to eliminate reactive oxygen species and regulate critical cell signaling pathways, such as nuclear factor-kappa B (NF-κB) and activator protein 1 (AP-1) [6]. These mechanisms confer substantial health benefits and have positioned TP as promising candidates for managing inflammation-related diseases, particularly psoriasis. Despite their therapeutic potential, TP face several challenges, including low bioavailability, instability under diverse conditions, and limited membrane permeability, which collectively restrict their effectiveness in clinical applications [7]. Addressing these limitations requires innovative delivery strategies to optimize their stability, absorption, and therapeutic impact.

As a medium-chain fatty acid mainly present in coconut oil, lauric acid (LA) has been approved by the FDA as a safe food additive [8]. It is also widely used in cosmetics, as noted in the Catalogue of Used Cosmetic Raw Materials (Edition 2021). Our previous research demonstrated its efficacy against both aerobic and anaerobic pathogens [9]. The antimicrobial efficacy of lauric acid is primarily attributed to its ability to disrupt the cell membranes of bacteria, resulting in cell lysis and death [10]. With its unique structure, LA can seamlessly integrate into the phospholipid bilayer of liposomes as a fatty acid chain [11]. This incorporation endows the liposomes with distinctive antibacterial properties. Given the advantages of TP and LA, we investigated the incorporation of TP into LA-integrated liposomes (LA-Lipo) as a promising approach to the topical therapeutic strategy of psoriasis. This strategic combination aims to utilize the beneficial properties of both compounds while addressing the challenges presented by the inherent limitations of TP, such as low bioavailability and instability.

Here, we constructed a sprayable hydrogel incorporating TP-loaded LA-Lipo (TP@LA-Lipo gel) for efficient anti-inflammation and antibacterial to address the problem of psoriasis. Sprayable hydrogel delivering TP@LA-Lipo not only facilitates ease of application but also provides sustained release of liposomes over time. This formulation enables TP@LA-Lipo to penetrate the deeper layers of the skin, enhancing its ability to target inflammatory mediators. Notably, TP@LA-Lipo exhibits antibacterial effects by disrupting the bacterial membrane, while simultaneously reducing inflammation-related factors, such as TNF-α and iNOS, in macrophages. As proof of principle, we evaluated the therapeutic efficacy of TP@LA-Lipo gel in an animal model of psoriasis. Our results demonstrated that TP@LA-Lipo gel effectively normalized the skin lesions in the psoriasis area, reducing the frequency of reapplications and improving the overall management of psoriasis symptoms. While most therapeutic approaches for psoriasis focus on inhibiting inflammatory cytokines, many of these treatments may inadvertently promote bacterial growth, allowing for persistence within the host [12]. This study uniquely addresses the bacteria-induced itch-scratch-inflammation cycle by simultaneously targeting bacterial infection and inflammation using TP@LA-Lipo gel, offering valuable insights into this dual therapeutic approach.

## 2. Materials and Methods

### 2.1. Materials

Lecithin with a purity of 99.8% and cholesterol with a purity of 99% were commercially acquired from Shanghai Aladdin Biochemical Technology Co., Ltd., located in Shanghai, China. Tea polyphenol (98%; Catalog # 84650-60-2) used in this research was purchased from Shanghai Yuanye Bio-Technology Co., Ltd., located in Shanghai, China. Rhodamine B (RhB) was purchased from Tianjin Damao Chemical Reagent Partnership Enterprise (Tianjin, China). Lauric acid (LA) was purchased from Macklin Reagent Company, located in Shanghai, China. Hero ink #201, purchased from Shanghai Hero (Group) Co., Ltd. (Shanghai, China). The strains *Escherichia coli* (ATCC 25922) and *Staphylococcus aureus* (ATCC 25923) were obtained from the American Type Culture Collection (ATCC, Manassas, VA, USA). RAW264.7 and HaCaT cell lines were obtained from the American Type Culture Collection. All the other reagents were commercially available and used as received. Vital River Laboratory Animal Technology Co., Ltd. (Beijing, China) supplied female BALB/c mice that were 6–8 weeks old and weighed between 16 and 18 g. Imiquimod (IMQ, 5%, *w*/*w*) was commercially acquired from Hubei Keyi Pharmaceutical Co., Ltd., located in Beijing, China. All animals were housed under standard conditions and no limitation of food or water. The temperature of animal room is 23 ± 1 °C and the humidity is 35–55%. All mice used in this study were euthanized via cervical dislocation under deep anesthesia at the end of experiments.

### 2.2. Physicochemical Characterization

#### 2.2.1. Preparation of LA-Lipo and TP@LA-Lipo

LA-Lipo and TP@LA-Lipo were prepared following a previously reported protocol [13]. Briefly, lecithin, cholesterol, lauric acid, and tea polyphenols were dissolved in ethanol at room temperature in a mass ratio of 20:5:1:4 and quickly injected into the same volume of phosphate-buffered saline (PBS; pH 6.0, 0.05 M). Then, it was stirred vigorously and kept for half an hour. The liposomal system was transferred to a rotary evaporator and lowered into a 45 °C water bath. Ethanol was removed under reduced pressure during the hydration reaction. TP@LA-Lipo obtained were applied to a high-pressure homogenizer (NanoGenizer 30k, Genizer, LA, USA) at room temperature. The parameter of the high-pressure homogenizer is 20 kpsi. Similarly, blank liposomes without drug loading (Lipo) or RhB-loaded LA-Lipo (RhB@LA-Lipo) were prepared using the same protocol. Finally, all prepared samples were stored at 4 °C until use.

#### 2.2.2. Characterization of TP@LA-Lipo

Dynamic light scattering measurements (Malvern Panalytical ZSU3100, London, UK) were used to test the particle size and zeta potential of TP@LA-Lipo and Lipo. Each experiment was performed in triplicate (*n* = 3). Morphological characterization of TP@LA-Lipo was carried out via transmission electron microscopy (Talos F200X G2, Thermo Fisher Scientific, Brno, The Czech Republic). A drop of liposomes (100-fold dilution) was deposited onto a lacey carbon film grid and left for 1 min. The excess solution was removed with filter paper and dried naturally at room temperature [14].

#### 2.2.3. Efficiency of LA Loading

LA was replaced by NR to determine the efficiency of LA loading. Nanoparticle Tracking Analysis (NanoSight Pro, Malvern, London, UK) was used to determine the concentration of LA-modified liposomes and total liposomes [15]. All samples were diluted in PBS and the final volume was 1 mL. Find the ideal measurement concentration by pre-testing the ideal particle per frame value. All settings of the experiments were set according to the manufacturer’s software manual (NanoSight Pro System User Guide, MAN066661-01-EN, 2023). Experiments were performed in triplicate. The efficiency of LA loading was calculated as follows:

(1) $$\mathrm{Efficiency}\;\mathrm{of}\;\mathrm{LA}\;\mathrm{Loading}\;(\%)=\frac{\mathrm{concentration}\;\mathrm{of}\;\mathrm{fluorescent}\;\mathrm{particles}}{\mathrm{concentration}\;\mathrm{of}\;\mathrm{non}\text{-}\mathrm{fluorescent}\;\mathrm{particles}}\;\times 100$$

#### 2.2.4. Determination of Percent Drug Encapsulation Efficiency

An ultraviolet spectrophotometer (UV-2600, Shimadzu, Kyoto, Japan) was used to create a standard calibration curve of TP. TP@LA-Lipo was performed at 12,000 rpm for 30 min, and then the TP concentration was calculated based on the value at 276 nm. The encapsulation efficiency (EE) of TP-loaded liposomal formulations was calculated by the following formula:

(2) $$\mathrm{EE}\;(\%)=\frac{\mathrm{Initial}\;\mathrm{TP}\;\mathrm{concentration}\text{-}\;\mathrm{concentration}\;\mathrm{of}\;\mathrm{TP}\;\mathrm{in}\;\mathrm{supernatant}}{\mathrm{Initial}\;\mathrm{TP}\;\mathrm{concentration}}\;\times 100$$

#### 2.2.5. Preparation of TP@LA-Lipo Gel

The “cold method” was adopted to prepare the P407 hydrogel vehicle. Specified amounts of P407 (15%/25%/35% *w*/*v*) and liposomal formulations were mixed with magnetic stirrers for 24 h at 4 °C until a homogeneous dispersion was obtained. The samples were stored at 4 °C until use. In the gel, the final concentration of TP@LA-Lipo is equivalent to 1 mg/mL of LA and 4 mg/mL of TP.

#### 2.2.6. Sprayability

A TP@LA-Lipo gel solution (3 mL) was prepared with red ink (20 μL) as a visual tracer. The solution was loaded into a sprayer with a 1 mm nozzle diameter and sprayed onto A4 paper and the skin of an arm, maintaining a 10 cm nozzle-to-target distance. The resulting spray patterns were photographed using a digital camera. The measurements were performed in triplicate.

#### 2.2.7. In Vitro Hydrogel Erosion

Hydrogel erosion was assessed following a previously reported protocol [16]. Briefly, TP@LA-Lipo gel and blank gel (25% *w*/*v*, 400 μL) were first weighed after gelation at 35 ± 0.5 °C (W_0_). Afterward, 300 μL of PBS with a pH of 7.4 was applied to the hydrogel surface, which was then incubated at 35 ± 0.5 °C. The weight of the hydrogel was measured at regular time intervals after blotting the excess surface water with filter paper carefully (W_t_). Erosion study was performed in triplicate. The percentage of the remaining hydrogel was calculated using the following equation:

(3) $$\mathrm{Weight}\;\mathrm{of}\;\mathrm{remaining}\;\mathrm{hydrogel}\;(\%)=w_{t}/w_{0}\times 100$$

where W_t_ and W_0_ are the weights of the remaining hydrogel and initial hydrogel, respectively.

#### 2.2.8. Rheological Properties

A rheometer (HAAKE MARS, Thermo Fisher Scientific, Karlsruhe, Germany) was employed to assess the rheological characteristics of the TP@LA-Lipo gel. The TP@LA-Lipo gel was loaded between the base plate (35 mm in diameter) and the rotor of a rheometer. The gap size used in this study was 0.4 mm. The oscillation temperature sweeps of TP@LA-Lipo gel performed under different temperatures at a frequency of 1.0 Hz. The temperature at which storage modulus (G′) and loss modulus (G″) were equal was identified as the gelation temperature of the TP@LA-Lipo gel [17]. To assess variations in the G′ and G″, frequency scans ranging from 0.01 to 10 Hz were conducted under a constant shear strain of 1%. Three-interval thixotropy tests were conducted at 35 °C. The shear rate of interval 2 is 10 s^−1^. The duration of each time interval was 180, 5, and 180 s. All tests were performed in triplicate.

### 2.3. In Vitro Biopharmaceutical Studies

#### 2.3.1. Microbiological Environment of the Psoriatic Skin

BALB/c mice (female, 16–18 g, 6–8 weeks) were anesthetized and the dorsal hair was removed. The BALB/c mice were then separated into two groups, each containing three mice (*n* = 3). One group was healthy BALB/c mice. The other group was psoriatic model constructed by IMQ. Isolated skin tissues of psoriatic mice and healthy mice were homogenized in 5 mL PBS. The mixtures were spread on the agar plate at a suitable dilution (1000×) with sterilized PBS and subsequently cultured at 37 °C for 24 h. The resulting photographs were taken using a digital camera [18,19].

#### 2.3.2. Cellular Uptake Test

The intracellular uptake behavior of TP@LA-Lipo in RAW264.7 cells was investigated quantitatively using flow cytometry (CytoFLEX, Beckman Coulter, Miami, FL, USA) as reported [20]. In short, RAW264.7 macrophage cells, at a density of 1×10⁶ cells per well, were plated in 6-well plates and maintained at 37 °C in a 5% CO₂ atmosphere with DMEM containing 10% FBS. The cells were treated with RhB@LA-Lipo and RhB solution at the same RhB concentration (2 μg/mL) for 4 h, respectively. Following incubation, the culture medium was discarded, and the RAW264.7 cells were collected. They were then rinsed three times with cold PBS and suspended in PBS for further analysis using flow cytometry. Tests were performed in triplicate.

#### 2.3.3. RT-qPCR Assay

RAW264.7 cells were seeded onto a 24-well plate in DMEM/10% FBS and incubated in a CO_2_ incubator until reaching 60–80% confluence. The cells were rinsed with PBS, and the medium was replaced with DMEM/10% FBS containing the TP@Lipo (200 μg/mL), LA-Lipo (50 μg/mL), and TP@LA-Lipo (200 μg/mL TP, 50 μg/mL LA) in the presence of LPS (1 μg/mL) for 24 h. RNA of RAW264.7 cells was extracted using RNAiso Plus (9109; TaKaRa). RNA was reverse-transcribed by ABScript™ RT Master Mix (RK20433, ABclonal). The real-time PCR analysis was conducted with TB Green Premix Ex Taq II (RR820A, TaKaRa) in a real-time PCR Detection System (LightCycler® 480 Instrument II, Roche, Zug, Switzerland). GAPDH was chosen as a housekeeping gene. The data were normalized using ΔΔCt method. The detailed primer sequences of mouse primers are as Table 1:

#### 2.3.4. In Vitro Antibacterial Assays

*S. aureus* and *E. coli* were used to evaluate antibacterial activity in this study. The bacteria were cultured in LB medium at 37 °C for a period of 16 to 18 h in an orbital shaker at 200 rpm and the culture was transferred to LB agar plate. In each experiment, a single colony of *S. aureus*, and *E. coli* was picked from the LB agar plate and cultured at 37 °C, 200 rpm in LB medium for 12 h. The fresh bacterial culture (2 mL, 1 × 10^6^ CFU/mL) containing the TP@Lipo (200 μg/mL), LA-Lipo (50 μg/mL), and TP@LA-Lipo (200 μg/mL TP, 50 μg/mL LA) were added in a sterile culture tube (*n* = 5). The mixture was incubated at 37 °C with constant agitation at 200 rpm. At 0, 4, 8, and 24 h, bacterial growth in the culture media was measured at 600 nm using an ultraviolet spectrophotometer (UV-2600, Shimadzu, Kyoto, Japan) at 600 nm (OD600) [21]. And the growth multiples of *S. aureus* and *E. coli* in each group at each time point were calculated according to the following formula:

(4) $$\mathrm{Bacteria}\;\mathrm{growth}\;\mathrm{rate}=\frac{OD600(t_{n})}{OD600(t_{0})}$$

where OD600 (t_0_) is the absorbance at 600 nm of each well at 0 h, and OD600 (t_n_) is the absorbance at 600 nm of each well at each time point.

#### 2.3.5. In Vitro Antibiofilm Assay

Additionally, the impact of TP@Lipo, LA-Lipo, and TP@LA-Lipo on bacterial biofilms (*S. aureus* biofilm and *E. coli* biofilm) underwent assessment. The application of TP@Lipo, LA-Lipo, and TP@LA-Lipo to biofilms, which were cultivated in 24-well plates, adhered to a specific treatment protocol [22]. Afterwards, the growth medium was discarded, and the biofilms were washed three times with PBS. Methanol was used to fix the biofilm for 15 min. After the methanol was removed, the resulting sediment was allowed to dry naturally. Subsequently, a staining procedure with 0.1% crystal violet solution was carried out for 25 min. To remove excess dye, the biofilms were washed three additional times with PBS. The petri dishes, placed upside down on filter paper, were used to remove moisture. Once the Petri plates were dry, a solution of 33% acetic acid was applied to dissolve the crystal violet, maintained at 37 °C for 30 min, before measuring the absorbance at 590 nm. Each assay was performed in triplicate.

#### 2.3.6. Zeta Potential Analysis of Bacterial Membrane Integrity

The integrity of the cell membrane was evaluated prior to and following liposome treatment by determining the cell surface charge using a zeta potential analyzer [23]. To mitigate concerns regarding potential interference from the zeta potential of liposomes, centrifugation was employed to remove the liposomes present in the supernatant, while the precipitated bacteria were collected as described in the literature [24]. Centrifugation of the bacterial samples was carried out at 4000 rpm for a duration of 5 min, after which the supernatant was discarded, and the cell pellets were washed with PBS (pH 7.4) five times. A bacterial cell suspension was then formulated by re-suspending the cell pellet in PBS (pH 7.4). The zeta potential was gauged using a Zetasizer device (Malvern Panalytical ZSU3100, London, UK). To ensure reproducibility, the experiment was performed in triplicate.

#### 2.3.7. Skin Permeation of TP@LA-Lipo In Vivo

BALB/c mice (female, 16–18 g, 6–8 weeks) were anesthetized, and the dorsal fur was removed. The BALB/c mice were used to establish an IMQ-induced psoriatic model. A vessel was fixed onto the back area using an adhesive. Subsequently, 20 μL of RhB, RhB@LA-Lipo, and RhB@LA-Lipo gel containing equivalent amounts of RhB were added to the containers of the three groups (*n* = 3). Following 4 h of permeation in a dark environment, the skin that had been permeated was scanned in a stratified manner by confocal laser scanning microscope (FV3000, OLYMPUS, Tokyo, Japan) [25].

### 2.4. Preclinical In Vivo Studies

#### 2.4.1. Animals and Psoriasiform Model

An IMQ-induced psoriasis model was developed using female BALB/c mice weighing 18–20 g and aged 6–8 weeks. The fur on the dorsal skin was shaved, and the area (2.5 cm^2^) was treated with a commercial depilatory cream (Veet^®^, Jingzhou, China). After a 24-hour recovery period, 125 mg of IMQ cream was applied topically to the shaved area daily for 7 consecutive days. This experimental procedure effectively induces psoriasis-like skin inflammation in mice in a reproducible manner [26].

The mice were randomly allocated into four groups (three per group): (1) Control: healthy mice; (2) IMQ: IMQ-induced mice receiving topical normal saline; (3) IMQ + TP@LA-Lipo: IMQ-induced mice receiving a topical TP@LA-Lipo solution; (4) IMQ + TP@LA-Lipo gel: IMQ-induced mice receiving TP@LA-Lipo gel.

#### 2.4.2. Psoriasis Area and Severity Index (PASI)

The PASI score was assessed according to the severity of the p psoriasiform skin lesions from day 1 to day 8. Erythema, scaling, and thickening were independently scored ranging from 0 to 4 as follows: 0 for none, 1 for mild, 2 for moderate, 3 for marked, and 4 for very evident. The total score represented the severity of inflammation, ranging from 0 to 12 [27].

#### 2.4.3. Changes of Spleen in Mice

The mice of the four groups were weighed prior to sacrificed, and their spleens were subsequently weighed to calculate the spleen index, reflecting the dynamic body changes.

#### 2.4.4. Histological Analysis

Following distinct interventions, dorsal skin tissues were gathered at predetermined intervals and preserved using a 4% paraformaldehyde solution. These skin tissues were processed for hematoxylin-eosin (H&E) staining. All prepared tissue sections were examined under a microscope (BioTek Cytation 5, Agilent Technologies, Santa Clara, CA, USA). The histopathological assessment in the psoriasiform model was based on the Baker grading system as described in a prior study [25].

### 2.5. Statistical Analysis

Analyses of statistics were conducted with Prism 9.0 software (GraphPad Software), using unpaired Student’s *t*-tests and one-way or two-way ANOVA, complemented by post hoc Bonferroni multiple comparisons. The datasets were roughly normally distributed with similar variances with similar differences between groups. Statistical significance is denoted at the following levels: n.s. when *p* > 0.05, * for *p* < 0.05, ** for *p* < 0.01, *** for *p* < 0.001, and **** for *p* < 0.0001.

## 3. Results and Discussion

### 3.1. The Synthesis and Characterization of TP@LA-Lipo

**Figure 1:**
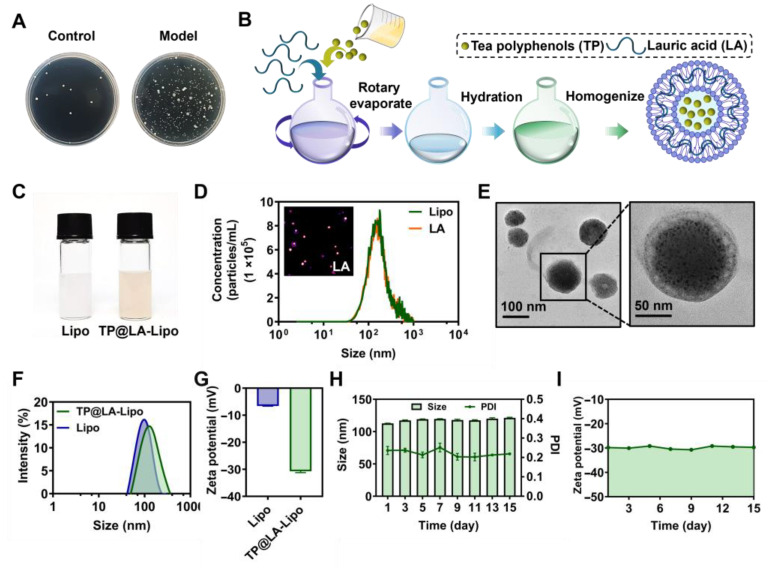
The fabrication and characterization of TP@LA-Lipo. (**A**) Bacterial colonies of cultures from normal skin and psoriatic skin tissues. (**B**) Schematic illustration of the construction of TP@LA-Lipo. (**C**) Digital picture of the Lipo and TP@LA-Lipo. (**D**) The concentrations of the non-fluorescent and fluorescent particles were measured by Nanosight particle tracking analysis. (**E**) TEM image of TP@LA-Lipo. (**F**) Particle size distribution of TP@LA-Lipo and Lipo. (**G**) Zeta potential of TP@LA-Lipo and blank Lipo. (**H**) The alterations in particle size and PDI of TP@LA-Lipo (*n* = 3). (**I**) Changes in zeta potential of TP@LA-Lipo (*n* = 3).

Psoriasis is characterized by an abnormal immune response and chronic inflammation, which weakens the skin’s barrier function, making it more susceptible to bacterial infections [28,29]. Increasing evidence suggests that various bacteria play key roles in the induction and exacerbation of psoriasis [30,31]. Our data also indicate that the bacterial load in psoriatic lesions is generally higher than that in normal skin, suggesting that psoriatic skin harbors a greater bacterial burden compared to healthy skin (Figure 1A).

To eliminate bacteria on psoriatic skin and promote its recovery, we selected LA, a saturated fatty acid with a 12-carbon atom chain, to impart antibacterial properties to liposomes. With its hydrophobic tail, LA can easily integrate into the hydrophobic region of the liposome’s phospholipid bilayer, essentially becoming part of the liposome’s membrane structure [11]. This integration allows for the delivery of LA alongside the anti-inflammatory therapeutic payload, TP, encapsulated within the liposomes. The preparation of LA-Lipo and TP loading was conducted using the well-established ethanol injection method [13] (Figure 1B). All formulated preparations were characterized for their physical properties and TP loading, and their stability was verified. Unlike the milky white appearance of blank liposomes, TP@LA-Lipo exhibits a slight tea color (Figure 1C). To evaluate the efficiency of LA loading, we used NanoSight Pro to detect the percentage of LA-Lipo among all liposomes. Due to challenges in directly detecting LA, NR, a dye with comparable lipid solubility, was used as a proxy for LA during this quantification. The proportion of fluorescent nanoparticles to the total number of nanoparticles was above 98%, indicating that almost all liposomes were modified (Figure 1D). Meanwhile, the encapsulation efficiency of TP@LA-Lipo was evaluated, with a determined value of 36.60 ± 3.10%. Transmission electron microscopy (TEM) revealed that TP@LA-Lipo exhibited a uniform cup-shaped morphology with an average particle size of 104.04 ± 18.09 nm. (Figure 1E). Dynamic light scattering measurements indicated that TP@LA-Lipo had a larger average size of 112.63 ± 0.42 nm, compared to the blank liposomes (Lipo), which measured 93.07 ± 0.78 nm (Figure 1F). This finding is consistent with the results obtained from the TEM analysis. Zeta potential measurements further demonstrated that TP@LA-Lipo exhibited a surface charge of −30.71 ± 0.50 mV, which was significantly lower than the −6.58 ± 0.07 mV observed for Lipo. (Figure 1G). This difference is attributed to the deprotonation of lauric acid’s carboxyl group during liposome formation. The more negative zeta potential enhances TP@LA-Lipo stability by reducing electrostatic repulsion between liposomes, thereby minimizing aggregation [32]. Additionally, TP@LA-Lipo maintained stable size, polydispersity index (PDI), and zeta potential throughout a fortnight (Figure 1H,I). These results indicate that TP@LA-Lipo possesses favorable physical properties for incorporation into hydrogels [33].

### 3.2. In Vitro Analysis of TP@LA-Lipo’s Antibacterial Activity

**Figure 2:**
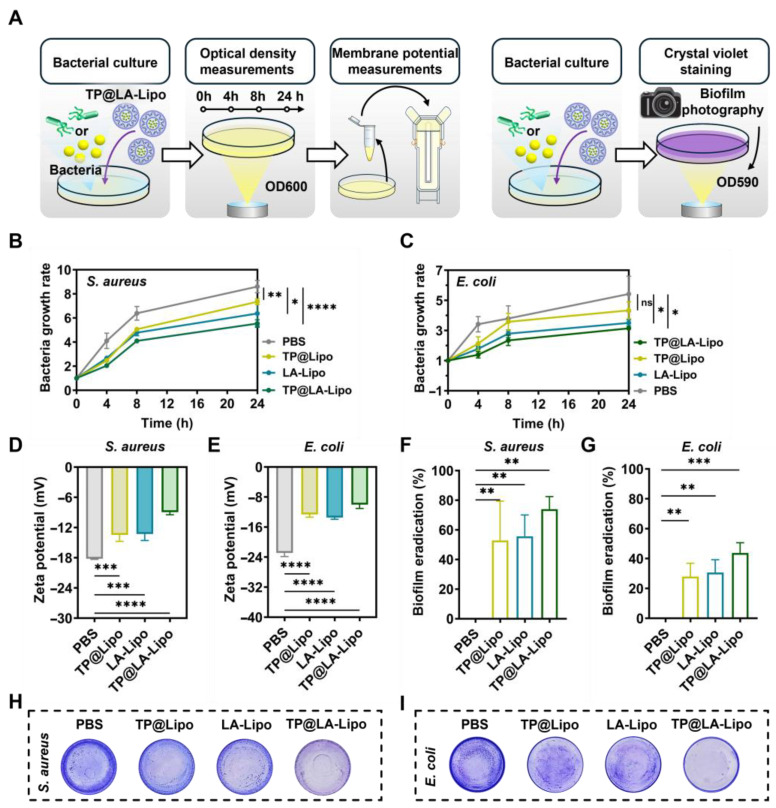
Antibacterial activity of TP@LA-Lipo on *S. aureus* and *E. coli* in vitro. (**A**) Schematic diagram of the antibacterial tests in vitro. (**B**) Bacterial growth rate of *S. aureus* with different treatments (*n* = 5). (**C**) Bacterial growth rate of *E. coli* with different treatments (*n* = 5). (**D**) Zeta potential of *S. aureus* processed with different treatments (*n* = 3). (**E**) Zeta potential of *E. coli* processed with different treatments. (**F**) Biofilm eradication of *S. aureus* biofilm after various treatments (*n* = 3). (**G**) Biofilm eradication of *E. coli* biofilm after various treatments (*n* = 3). (**H**) The photographs of *S. aureus* biofilm processed with different treatments and subjected to crystal violet staining. (**I**) The photographs of *E. coli* biofilm processed with different treatments and subjected to crystal violet staining. The data are shown as mean ± SD; * *p* < 0.05, ** *p* < 0.01, *** *p* < 0.001, **** *p* < 0.0001 and ns *p* > 0.05 via one-way ANOVA test or two-way ANOVA test.

Subsequently, the antimicrobial activity of TP@LA-Lipo against psoriasis was evaluated, particularly targeting Gram-positive *S. aureus* and Gram-negative *E. coli* [34]. Growth curves, biofilm assay, and membrane potential measurements were used as indices to assess the antibacterial activity of TP@LA-Lipo (Figure 2A). First, we evaluated the antibacterial efficacy of TP@LA-Lipo against *S. aureus* and *E. coli*. The results indicated that TP@LA-Lipo had comparable effects on both bacterial strains (Figure 2B,C). Specifically, at 24 h post-exposure, TP@LA-Lipo resulted in an approximate 27.78% reduction in *E. coli* and 29.88% reduction in *S. aureus* compared to the negative control. While TP@Lipo and LA-Lipo also demonstrated inhibitory effects on the growth of both bacterial strains, TP@LA-Lipo exhibited superior antibacterial activity during the 24-h assessment. These findings suggest that *E. coli* and *S. aureus* are more susceptible to TP@LA-Lipo than to TP@Lipo and LA-Lipo. Then, we analyzed changes in membrane potential to investigate whether TP@LA-Lipo disrupts the bacterial cell membrane. As shown in Figure 2D,E, bacteria exposed to TP@LA-Lipo showed a marked increase in membrane potential. This suggests that TP@LA-Lipo treatment caused damage to the bacterial cell membrane, which may destroy the cell integrity, and increase leakage of intracellular material [35,36]. Encouraged by the advantages of TP@LA-Lipo, we next investigated its efficacy in eradicating biofilms formed by *S. aureus* and *E. coli*, which are closely related to bacterial pathogenicity. The capacity of the TP@LA-Lipo to reduce biofilm formation was evaluated by crystal violet staining. As depicted in Figure 2F–I, the biomass of the adhered biofilm in the three treated groups was significantly lower than that in the untreated group, with TP@LA-Lipo demonstrating the most pronounced anti-biofilm effect, inhibiting 74.07% (*S. aureus*) and 43.76% (*E. coli*) of biofilm formation.

### 3.3. Anti-Inflammatory Effects of TP@LA-Lipo on Macrophages

**Figure 3:**
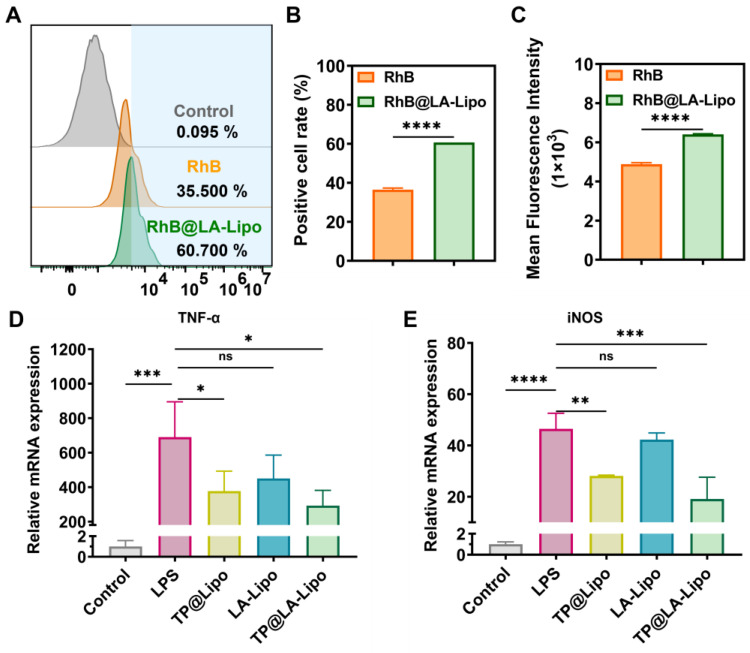
TP@LA-Lipo enhanced cellular uptake and reduced inflammation. (**A**) The uptake of TP@LA-Lipo by RAW264.7 cells following a 4-hour incubation period is depicted in the flow cytometry diagrams. (**B**) Percentage of fluorescence-positive cells after treatments with RhB@LA-Lipo and RhB solution (*n* = 3). (**C**) Mean fluorescence intensity of positive cells was determined by flow cytometry (*n* = 3). (**D**,**E**) RT-qPCR results showing the representative gene expression levels (*n* = 3). All data are expressed as mean ± SD. Significance is indicated as the following levels: * for *p* < 0.05, ** for *p* < 0.01, *** for *p* < 0.001, **** for *p* < 0.0001, and ns *p* > 0.05 via Student’s *t*-tests or one-way ANOVA test.

Macrophages, which are abundant in the epidermal tissue, are critical inflammatory cells involved in psoriasis [37]. They can alter their phenotype, assuming either an M1 type that promotes inflammation or an M2 type that helps to alleviate the disease [38]. The effective delivery of TP into macrophages is crucial, as it significantly influences the anti-inflammatory outcomes in the treatment of psoriasis. Figure 3A–C shows that the liposome formulation, RhB@LA-Lipo, significantly increased RhB uptake, resulting in a 1.66-fold enhancement compared to RhB alone. The cytotoxicity tests were conducted before performing RT-qPCR and the cell viability of RAW264.7 cells and HaCaT cells indicated the safety of TP@Lipo, LA-Lipo, and TP@LA-Lipo (Figure S2). In order to validate the anti-inflammatory properties of TP@LA-Lipo, RAW264.7 cells were stimulated with LPS to create an inflammation model, followed by quantification of the pro-inflammatory cytokines TNF-α and iNOS. The RT-qPCR assays indicated that treatment with TP@LA-Lipo significantly reduced the expression of TNF-α and iNOS mRNA in activated macrophages compared to LPS-activated controls. Of note, while TP@ Lipo also reduced the levels of TNF-α and iNOS, the effect of TP@LA-Lipo was markedly greater (Figure 3D,E), suggesting that TP@LA-Lipo possesses excellent anti-inflammatory properties.

### 3.4. Characterization of Sprayable Hydrogels Loaded with TP@LA-Lipo

**Figure 4:**
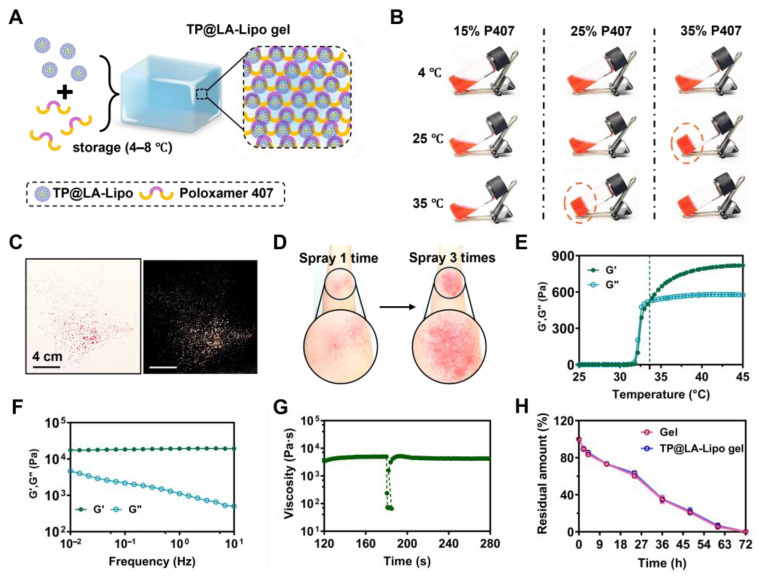
Preparation and characterization of TP@LA-Lipo gel. (**A**) Schematic illustration of TP@LA-Lipo. (**B**) Optical images of the solutions of P407 (15%/25%/35% *w*/*v*) at 4 °C, 25 °C and 35 °C. Screening of optimal gelation condition with various concentrations of components of P407. (**C**) Representative images of sprayed TP@LA-Lipo gel. (**D**) Representative images of sprayed TP@LA-Lipo gel once and three times on the arm. (**E**) At a frequency of 1.0 Hz, the progression of storage modulus (G′) and loss modulus (G″) across various temperatures was observed. (**F**) Frequency sweeps of TP@LA-Lipo gel. (**G**) Three-interval-thixotropy test of TP@LA-Lipo gel performed at 100% strain. (**H**) Quantification of gel and TP@LA-Lipo gel degradation dynamics rate.

To enhance the skin penetration and sustained application of TP@LA-Lipo, we embedded it in a sprayable hydrogel. Sprayable hydrogels, which can be quickly applied to cover the skin surface, offer several advantages over traditional hydrogels [39]. These sprayable formulations are portable and can be easily applied to irregularly shaped surfaces and specific locations without physical contact, thereby minimizing the risk of contamination or irritation to psoriatic skin [40]. Poloxamer 407 (P407), an FDA-approved temperature-sensitive polymer, can be formulated into a sprayable liquid solution that transitions into a gel upon contact with body temperature, making it particularly suitable for skin application [41]. Due to its gel-forming properties, P407 can also be utilized to deliver drugs topically, facilitating sustained release at psoriasis sites. Hence, we combined TP@LA-Lipo with P407 and incubated the mixture at 4 °C to form the TP@LA-Lipo gel (Figure 4A). To optimize the gelation conditions, we screened three different P407 concentrations (15%, 25%, and 35% *w*/*v*) using the tilt vial test. Gelation was assessed after standing for 10 s at 4 °C, 25 °C, and 35 °C (Figure 4B). The 25% concentration, which gelled only at body temperature, was selected for further experiments. The TP@LA-Lipo gel exhibited stable, even sprayability (Figure 4C). Notably, the gel formed a thin film upon spraying onto the skin, with just three sprays (Figure 4D) and demonstrated biosafety after application (Figure S1). To investigate the mechanical characteristics, we conducted rheological tests on the TP@LA-Lipo gel. The results of amplitude sweep tests showed that the TP@LA-Lipo gel maintained a higher G′ than G″ under small strain, and the following condition was chosen: a strain of 1% (Figure S3). The apparent viscosity of the TP@LA-Lipo gel solution increased with temperature rising (Figure S4). The temperature-sweep test revealed that the thermogelation temperature of TP@LA-Lipo gel was 33.62 °C (Figure 4E). Given that the exposed surface skin temperature can easily drop, having a thermogelation temperature below 37 °C is advantageous for achieving stable gelation [42]. At 35 °C, the storage modulus (G′) of TP@LA-Lipo gel consistently exceeded the loss modulus (G″) across the 0.01–10 Hz oscillation range, with both moduli remaining within the plateau period. This indicates that the TP@LA-Lipo gel maintained its gel-like state (Figure 4F). To further assess the gel-like properties, a three-interval-thixotropy test was performed, yielding a relative recovery rate of 90% (Figure 4G). Additionally, erosion study demonstrated that both the blank gel and TP@LA-Lipo gel were completely eroded within three days (Figure 4H), indicating the sustained release of TP@LA-Lipo.

### 3.5. Evaluating Drug Penetration of TP@LA-Lipo Gel in Psoriatic Skin

**Figure 5:**
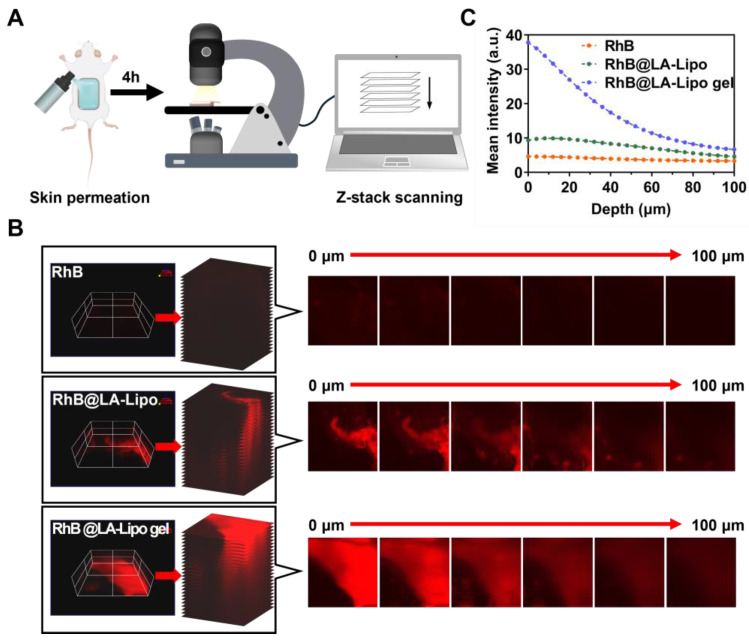
Enhanced permeation of RhB@LA-Lipo in vivo. (**A**) Schematic illustration of skin permeation assay. (**B**) Representative x-y-z orthogonal images of RhB, RhB@LA-Lipo, or RhB@LA-Lipo gel-treated skin and representative confocal images. (**C**) Mean fluorescence was quantified by ImageJ (2.15.1).

The stratum corneum, the outermost layer of the epidermis, becomes thickened and abnormally structured in psoriasis patients, making it challenging for drugs to penetrate and reach the inflamed layers beneath [18]. To evaluate the efficacy of drug penetration in psoriatic skin, we employed confocal laser scanning microscopy (CLSM) to analyze its distribution, with RhB serving as a fluorescence marker for TP. CLSM enabled the generation of detailed three-dimensional reconstructions of skins treated with RhB, RhB@LA-Lipo, or RhB@LA-Lipo gel (Figure 5A). Figure 5B showed the RhB fluorescence in skin slices following 4 h of in vivo permeation with the different formulations. Negligible fluorescence was observed in the RhB group, while noticeable fluorescence was evident on the skin surface treated with RhB@LA-Lipo. Notably, the RhB@LA-Lipo gel-treated skin exhibited significantly higher and deeper fluorescence intensity, detectable up to 100 μm. However, while the fluorescence intensity decreased with increasing skin depth and was consistently under 5.0 for the RhB group, the fluorescence signals in the RhB@LA-Lipo gel-treated skin remained above 20.0 at depths of approximately 30 μm (Figure 5C). These results suggest that when embedded in the hydrogel, the drug penetrates more deeply and efficiently.

### 3.6. Therapeutic Efficacy of TP@LA-Lipo Gel in IMQ-Induced Psoriasis Mouse Models

**Figure 6:**
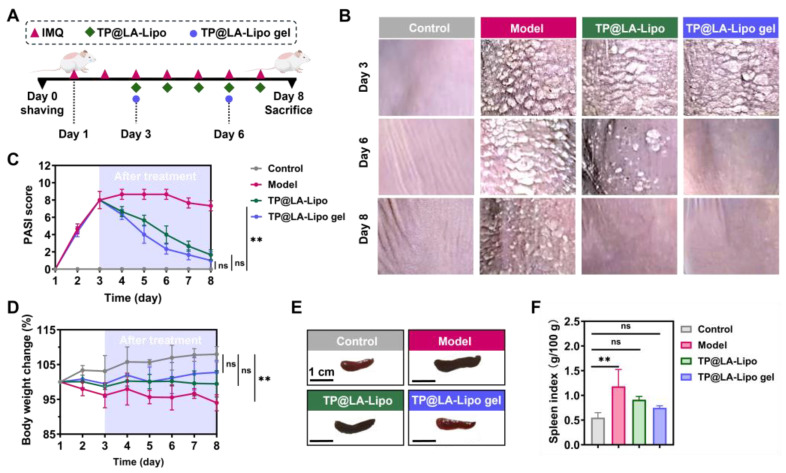
Transdermal therapy effects of TP@LA-Lipo and TP@LA-Lipo gel on imiquimod-induced psoriasis mice. (**A**) Experimental scheme of anti-psoriatic treatment. (**B**) Representative clinical features of psoriasis-like skin lesions throughout the experiment. (**C**) PASI scores of psoriatic skin lesions treated with different formulations (*n* = 3). (**D**) Change in body weight in percentage (*n* = 3). (**E**) Representative photographs of spleen of psoriatic mice treated with different formulations (*n* = 3). (**F**) Spleen index of psoriatic mice treated with different formulations (*n* = 3). The data are shown as mean ± SD; ** for *p* < 0.01 and ns *p* for >0.05 via one-way ANOVA test or two-way ANOVA test.

Given the significant antibacterial properties and the ability of TP@LA-Lipo to alleviate pro-inflammatory responses, along with the sustained release and improved penetration provided by the sprayable hydrogel formulation, we further evaluated the therapeutic efficacy of TP@LA-Lipo and TP@LA-Lipo gel in psoriasis mouse models. Psoriasis was induced in the mice by topical application of 5% IMQ cream, a toll-like receptor agonist that activates immune cells, as shown in Figure 6A. On day 3, when the mice exhibited inflamed and scaly lesions, they were treated with TP@LA-Lipo, TP@LA-Lipo gel, or saline. TP@LA-Lipo was administered daily, while TP@LA-Lipo gel was applied on days 3 and 6 (Figure 6A). The progression of psoriasis during treatment was assessed by photographing the lesions and measuring the PASI, which are widely used parameters for clinical evaluation [27]. Figure 6B,C showed that TP@LA-Lipo gel led to a more significant reduction in epidermal scaling and PASI scores compared to the untreated Model group (for a complete picture of the back skin, see Supporting Information Figure S5). Meanwhile, TP@LA-Lipo gel facilitated the shedding of nearly all epidermal scales by day 6 of treatment, whereas TP@LA-Lipo treatment led to complete shedding by day 8. Although TP@LA-Lipo also demonstrated a notable therapeutic effect, bringing the psoriatic skin close to a healthy state, TP@LA-Lipo gel not only reduced the dosing frequency but also facilitated a faster recovery. Furthermore, mice treated with TP@LA-Lipo gel showed the greatest increase in body weight, indicating superior efficacy compared to the other groups (Figure 6D). Considering the spleen plays a vital role in the immune system, its enlargement in the psoriasis model suggests a systemic immune response to skin inflammation. To assess the systemic effect of psoriasis, spleens were harvested from the treated mice on day 8, and the spleen index was calculated. As illustrated in Figure 6E,F, the spleen index for the model group was 1.18, reflecting an inflammatory response associated with the psoriasis model. After treatment, the TP@LA-Lipo group showed a reduction in the spleen index to 0.91, while the TP@LA-Lipo gel group exhibited a more significant decrease to 0.75. These findings indicated that TP@LA-Lipo gel demonstrated enhanced efficacy in modulating systemic inflammation.

**Figure 7:**
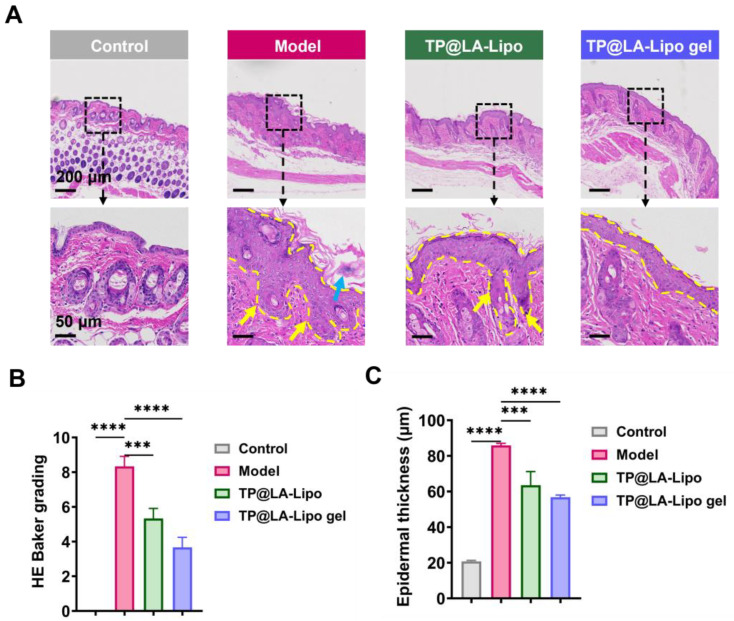
TP@LA-Lipo gel significantly ameliorated the psoriatic lesions of IMQ-induced mice. (**A**) Representative H&E images of the dorsal skin sections (The blue arrow marks Munro microabscesses, yellow arrows mark the acanthosis and the yellow dashed lines outline the stratum spinosum). (**B**) HE Baker grading of the dorsal skin sections stained with H&E (*n* = 3). (**C**) Quantification of the epidermal thickness (*n* = 3). *** for *p* < 0.001, **** for *p* < 0.0001 via one-way ANOVA test.

Histopathological analysis is essential for evaluating the effects of TP@LA-Lipo gel on psoriatic skin, providing detailed insights into structural changes and the inflammatory response. Thus, we performed skin histopathological examinations, with the results presented in Figure 7A. In the model group, a significant increase in epidermal thickness was observed, accompanied by severe desquamation and hyperkeratosis, resulting in a much thicker epidermis compared to normal skin. Furthermore, notable histological features such as acanthosis (indicated by a yellow arrow) and Munro microabscesses (indicated by a blue arrow) were also observed in the epidermis of the model group. Following treatment, TP@LA-Lipo gel exhibited the most remarkable reduction in epidermal thickness, and the HE Baker grading showed a significant decrease compared to the other groups (Figure 7B,C). These findings suggest that TP@LA-Lipo gel effectively reduces inflammatory skin changes, alleviates symptoms of psoriasis, and holds potential as a therapeutic option for managing this chronic skin disorder.

## 4. Conclusions

In summary, a novel antibiotic-free, sprayable hydrogel incorporating LA-modified liposomes loaded with TP was developed for the treatment of psoriasis. TP@LA-Lipo gel is sprayed onto the psoriasis lesions as a liquid, forming a hydrogel film that rapidly transitions into a semi-solid structure upon contact with body temperature. Subsequently, TP@LA-Lipo gel effectively eliminated both Gram-positive *S. aureus* and Gram-negative *E. coli* on the surface of psoriasis lesions. Following this, TP@LA-Lipo penetrated into the deeper layers of the skin, leading to a reduction in inflammation through the modulation of macrophage TNF-α and iNOS levels. In a psoriasis mice model, the TP@LA-Lipo gel markedly reduced both epidermal scaling and epidermal thickness, demonstrating significant improvements in skin health. Additionally, it promoted skin regeneration to a nearly healthy state and decreased spleen index. The sustained release of TP@LA-Lipo from sprayable hydrogel over three days maintained stable drug concentration, prolonging the therapeutic effect and reducing the frequency of administration. Therefore, considering its efficient inhibition of bacterial growth and modulation of inflammatory responses, TP@LA-Lipo gel could represent a safer and more effective therapeutic option for patients with psoriasis.

## Figures and Tables

**Table 1:** Primer sequences for RT-qPCR.

Gene	Forward Primer	Reverse Primer
GAPDH	GGTTGTCTCCTGCGACTTCA	TGGTCCAGGGTTTCTTACTCC
TNF-α	GGTGCCTATGTCTCAGCCTCTT	GCCATAGAACTGATGAGAGGGAG
iNOS	CACCTTGGAGTTCACCCAGT	ACCACTCGTACTTGGGATGC

## Data Availability

The original contributions presented in the study are included in the article and Supplementary Material, further inquiries can be directed to the corresponding authors.

## Supplementary Materials

The following supporting information can be downloaded at https://www.mdpi.com/article/10.3390/jfb16040124/s1, Figure S1: The biosafety of TP@LA-Lipo gel. Figure S2: Biosafety assessment of TP@LA-Lipo. Figure S3: The amplitude sweeps of TP@LA-Lipo gel at 35 °C with a constant frequency of 1.0 Hz. Figure S4: The viscosity-temperature curves of TP@LA-Lipo gel. Figure S5: Complete picture of the back skin.
