# Supplementary material for: A Tea Polyphenol-Infused Sprayable Thermosensitive Liposomal Hydrogel for Enhanced Anti-Inflammatory and Antibacterial Psoriasis Treatment
Source: J Funct Biomater. 2025 Apr 1;16(4):124. doi: 10.3390/jfb16040124 (PMC12027687; doi:10.3390/jfb16040124)
Supplement: Supplementary file 1 [file jfb-16-00124-s001.zip › jfb-3454257-supplementary.pdf]

# A Tea Polyphenol-Infused Sprayable Thermosensitive Liposomal Hydrogel for Enhanced Anti-Inflammatory and Antibacterial Psoriasis Treatment

Wei Shen [a,1], Qilian Ye [a,1], Hongbo Zhang [b], Shenghong Xie [a], Shiqi Xie [a], Cailian Chen [a], Jinying Liu [a], Zhengwei Huang [c], Hai-Bin Luo [a,*] and Ling Guo [a,*]

## S1. Materials

Dimethyl sulfoxide (DMSO) and 3-(4,5-dimethylthiazol-2-yl)-2,5-diphenyl tetrazolium bromide (MTT) were bought from Sigma-Aldrich (St. Louis, MO, USA). All the other reagents were commercially available and used as received.

## S2. Methods

*S2.1. Cytotoxicity assay*

RAW264.7 cells and HaCaT cells were seeded in 96-well plates (Costar, USA) at a density of 5,000 cells per well (100 µL per well), respectively. The medium was replaced with fresh medium containing TP@Lipo (200 µg/mL), LA-Lipo (50 µg/mL) and TP@LA-Lipo (200 µg/ mL TP, 50 µg/mL LA) when the cells reached approximately 80% confluence. After a further 24 h incubation period, MTT solution (20 µL, 5 mg/mL in PBS) was added to each well. Then the plates were incubated at 37°C for 4 h, until purple formazan crystals were clearly visible. The purple formazan crystals were dissolved by DMSO (150 µL per well). Absorbance was measured using a microplate spectrophotometer (Epoch, Bio-Tek, USA) at a wavelength of 570 nm. Tests were performed in triplicate.

*S2.2. Rheological Properties*

The rheological properties of TP@LA-Lipo gel were measured by a rheometer (HAAKE MARS, Thermo Fisher scientific, Germany). The TP@LA-Lipo gel was loaded between the base plate (35 mm in diameter) and a rotor of a rheometer. The gap size used in this study was 0.4 mm. The amplitude sweep test was implemented with 0.1–100 % shear strain at 35 °C with a constant frequency of 1.0 Hz. The temperature dependence of the complex viscosity was determined by oscillation temperature sweeps from 10 to 50 °C at a frequency of 1.0 Hz. All tests were performed in triplicate.

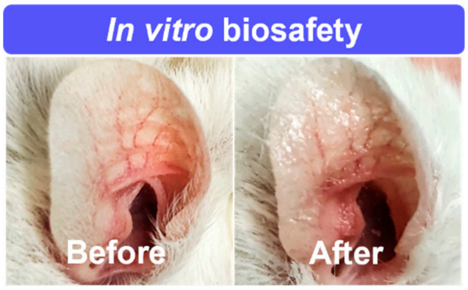


**Figure S1** The biosafety of TP@LA-Lipo gel.

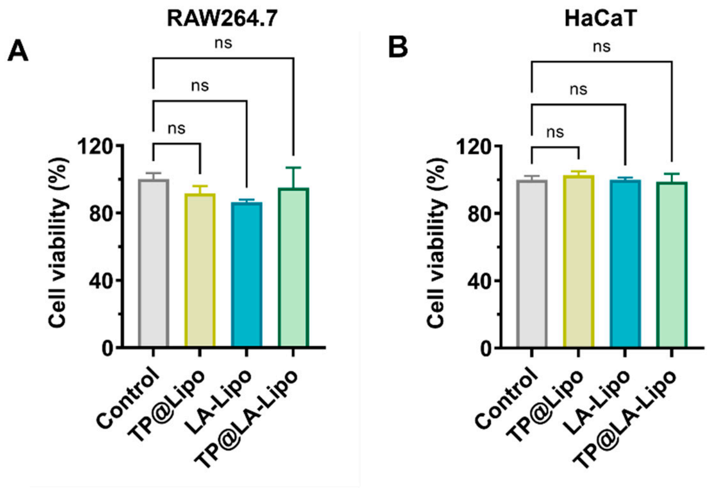


**Figure S2** Biosafety assessment of TP@LA-Lipo. (A) Cell viability of RAW264.7 cells treated with different formulations ($n$ = 3). (B) Cell viability of HaCaT cells treated with different formulations ($n$ = 3). The data are shown as mean ± SD; ns $p > 0.05$ via one-way ANOVA test.

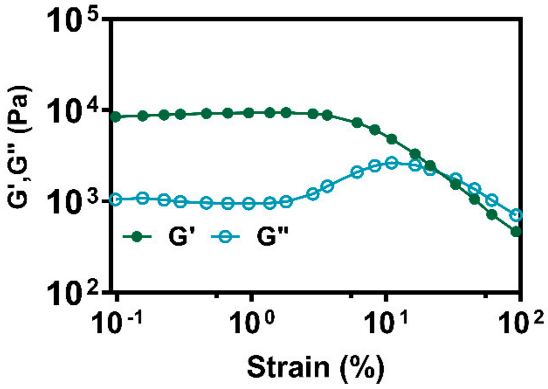


**Figure S3** The amplitude sweeps of TP@LA-Lipo gel at 35 ℃ with a constant frequency of 1.0 Hz.

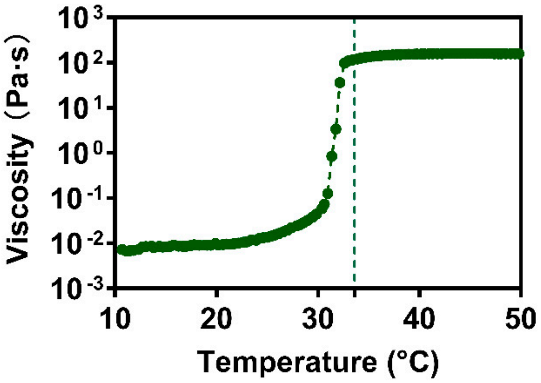


**Figure S4** The viscosity-temperature curves of TP@LA-Lipo gel.

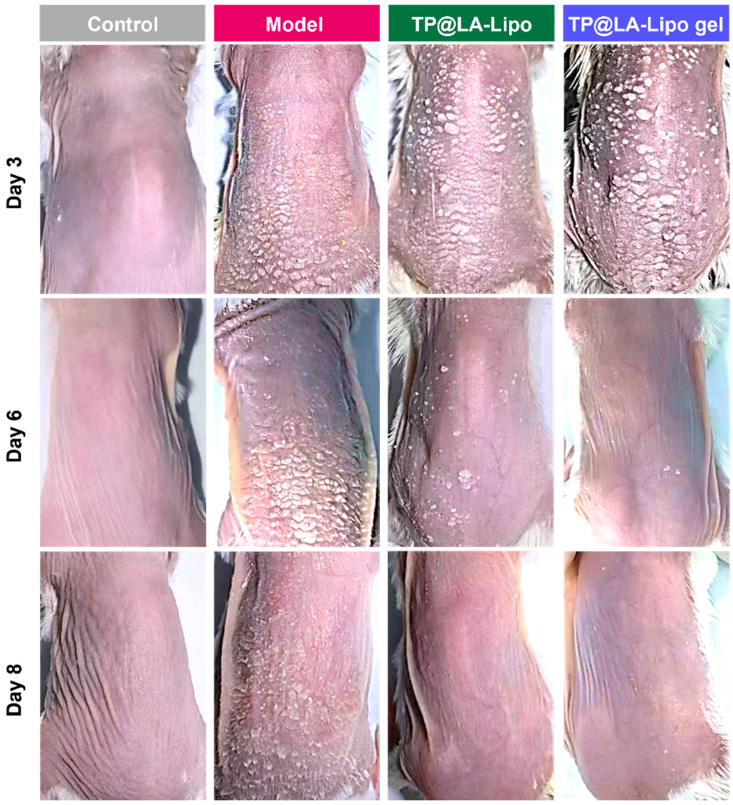


**Figure S5** Complete picture of the back skin.
